# Continuous Flow Microfluidic Bioparticle Concentrator

**DOI:** 10.1038/srep11300

**Published:** 2015-06-10

**Authors:** Joseph M. Martel, Kyle C. Smith, Mcolisi Dlamini, Kendall Pletcher, Jennifer Yang, Murat Karabacak, Daniel A. Haber, Ravi Kapur, Mehmet Toner

**Affiliations:** 1 BioMEMS Resource Center, Center for Engineering in Medicine and Surgical Services, Massachusetts General Hospital and Harvard Medical School, Boston, Massachusetts, 02114, USA; 2 Massachusetts General Hospital Cancer Center, Boston, Massachusetts, 02114, USA; 3 Shriners Hospital for Children, Boston, Massachusetts, 02114, USA

## Abstract

Innovative microfluidic technology has enabled massively parallelized and extremely efficient biological and clinical assays. Many biological applications developed and executed with traditional bulk processing techniques have been translated and streamlined through microfluidic processing with the notable exception of sample volume reduction or centrifugation, one of the most widely utilized processes in the biological sciences. We utilize the high-speed phenomenon known as inertial focusing combined with hydraulic resistance controlled multiplexed micro-siphoning allowing for the continuous concentration of suspended cells into pre-determined volumes up to more than 400 times smaller than the input with a yield routinely above 95% at a throughput of 240 ml/hour. Highlighted applications are presented for how the technology can be successfully used for live animal imaging studies, in a system to increase the efficient use of small clinical samples, and finally, as a means of macro-to-micro interfacing allowing large samples to be directly coupled to a variety of powerful microfluidic technologies.

From point-of-care diagnostics^1^ to massively parallelized arrays for gene expression detection^2^, microfluidic technologies are revolutionizing science and medicine. Due to the widespread study of physical phenomena in microfluidics and especially within biological systems, progress is being made towards accelerating drug discovery^3^, personalizing medical treatments^4^,^5^ and improving basic scientific studies of cells using the well-controlled experimental conditions on the scale of single cells^6^,^7^. Moreover, microfluidic devices are now being used in the processing of biological samples; for example, there are now microfluidic devices for isolating rare cells from blood replacing bulk isolation methods^8^,^9^,^10^,^11^. There are even new means of sorting cells on the microscale depending on specific secreted factors^12^.

While there are examples of cell lysis^13^, cell arrays^14^ and blood fractionation techniques^15^,^16^,^17^ being translated to the microscale, one of the fundamental macroscale processing techniques ubiquitously used in biological experiments has yet to be translated to the microscale; namely, volume reduction. Typically accomplished through centrifugation, reducing the volume of a suspension of bioparticles is used for exchanging buffer (removal of unwanted chemicals) and enriching a sample for downstream analyses. Centrifugation exposes bioparticles to heightened centrifugal forces for durations on the order of tens of minutes in order to form a compacted pellet at the bottom of a container. Excess fluid is then typically aspirated and the sample resuspended in a known volume manually, both inherently variable and low yield processes. The compaction into the pellet as a result of the centrifugal forces has also been shown to mechanically damage cells^18^ as well as alter gene expression^19^,^20^,^21^. While lower centrifugation forces minimize these harmful effects, the efficiency of the process is significantly compromised. Another technical challenge for centrifugation is dealing with large volume samples that require being split into several smaller fractions in order to be processed, increasing the risk of sample contamination as well as compounding losses. A continuous flow microfluidic device capable of reproducing the volume reduction process with high yield and integrity of the bioparticles will be a significant improvement over current techniques and has the potential to be integrated into many point-of-care systems and disseminated for global health diagnostics, application areas for which a typical centrifuge would not be ideal.

In this communication, we present a microfluidic device that couples the phenomenon of inertial focusing^22^,^23^ and highly controllable hydraulic resistance micro-siphoning channels to achieve controlled bioparticle manipulation and concentration^24^. Inertial focusing is the result of purely hydrodynamic forces, which in laminar flow can cause particles to migrate across streamlines to precise equilibrium positions. Generally, inertial focusing devices are operated in a manner such that particles focus to equilibrium positions and then flow streams containing particles of different sizes^25^, shapes^19^,^20^ and mechanical properties^26^,^27^ are separated into different outlets designed to split the flow^17^,^26^,^28^. We instead take advantage of the strong inertial forces adjacent to the walls of a microchannel which create a cell-free region that is then siphoned in a virtually continuous manner within an asymmetrically curved inertial focusing device^25^. By taking the concept of repetitive fluid removal and refocusing to an extreme, a parallelized and serially integrated version of our microfluidic concentration chip is able to reach a volume reduction factor >400 of a dilute cellular suspension with high yield, ~95%, at a throughput of 4 mL/min. We will present the design of this class of devices and explore its performance across the most pertinent variables for biological fluid processing.

## Results

### Design Concept

**Figure 1:**
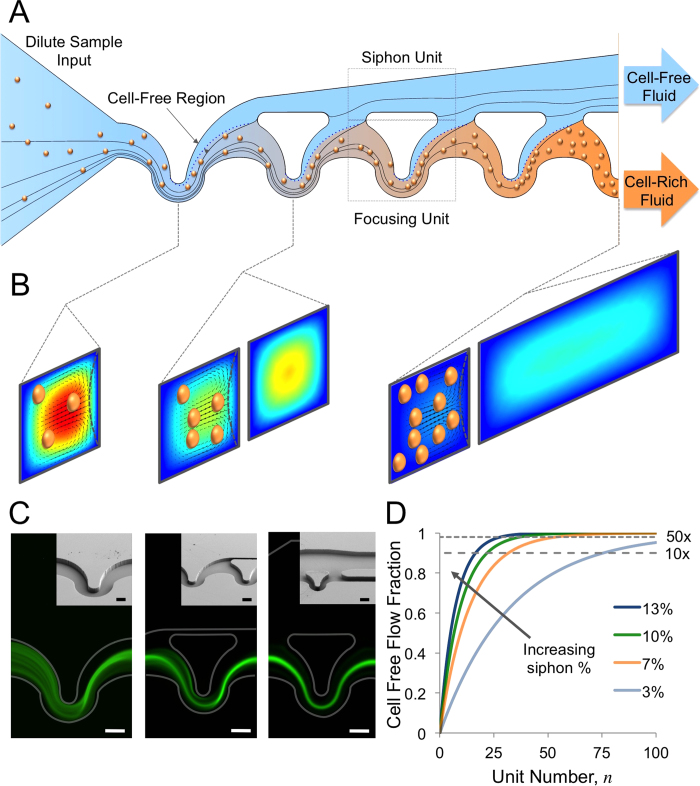
Microfluidic Volume Reduction Using Inertial Focusing and Continuous Siphoning: (**A**) Conceptual schematic of inertial focusing siphon sample volume reduction. Starting with a dilute suspension entering the device (i) particles move into the focusing units where the cell free region is formed (ii). By controlling the relative hydraulic resistances of the focusing units (bottom) and the siphon (top) this cell free region is shifted out of the focusing units (iii). The particles still on the focusing unit side form another cell free region in the next focusing unit that is consequently siphoned off (iv). This process is repeated until the desired volume reduction factor is achieved and the siphoned fluid and particle rich fluid leave the device via separate outlets (v). Lines indicate simulated 2D streamlines. (**B**) Flow simulation cross-sectional plots of the flow in through the inertial focusing siphon device showing the Dean flow vectors and velocity profile which causes the formation of the cell free region within the focusing units and that the overall flow speed decreases as fluid is siphoned off (red-high velocity to blue-low velocity). Particles are superimposed on the flow profiles. (**C**) A typical sample of fluorescently labeled buffy coat being run through a 10x volume reduction device at three different locations (1^st^ focusing unit, 1^st^ siphoning unit and outlet from left to right respectively) and scanning electron micrographs of the same locations. Scale bars are 50 μm. (**D**) A plot of the theoretical cell free fraction of the input fluid per unit, or along a device, with different siphon percentages used at each unit increasing from bottom to top (3%, 7%, 10% and 13%).

Briefly, the conceptual design of the inertial focusing based volume reduction devices is summarized in Fig. 1. A dilute suspension of particles or cells enters a device (Fig. 1A) which then accelerates as the channel converges and passes through asymmetrically curved inertial focusing channels that generate a cell free region on the top (opposite of the focusing units). This cell free region is then siphoned to an adjacent parallel channel opposite the focusing units. The amount of fluid siphoned is dependent on the relative hydraulic resistances of the siphon channel and the focusing channel. The cell rich fluid is then passed through another focusing channel creating a new cell free region that is then siphoned off. This repetitive process continues until the end of the device where the flows are split into calculated fractions coded by hydraulic resistors on each outlet. The flows within the focusing units and siphoning units are presented in Fig. 1B showing that in a typical device the flow speed and thus inertial forces will decrease along the length of the channel. Fluorescent streak images are presented in Fig. 1C showing the behavior of fluorescently labeled white blood cells through a characteristic device clearly showing that the cells are retained in the focusing channels at the outlets. By removing the same fraction of the volumetric flow rate through each consecutive focusing unit at each siphon the actual flux of fluid from the focusing channels into the cell free side channel decreases exponentially along the channel as shown in Fig. 1D. Higher siphon percentages decrease the overall number of units required to reach an equivalent volume reduction factor seen at the intersections of the 10x and 50x dashed lines and each of the different siphon percentage curves, which increase in siphon percentage from bottom curve to the top curve. The higher siphon percentages risk the loss of cells if the inertial forces do not create a large enough cell free region, thus illustrating the importance of the dynamics of the growth of the cell free region that would define an upper limit on the siphon percentage. A more detailed description of the design process is given in the Methods section.

### Cell Free Region Growth and Siphon Percentage

**Figure 2:**
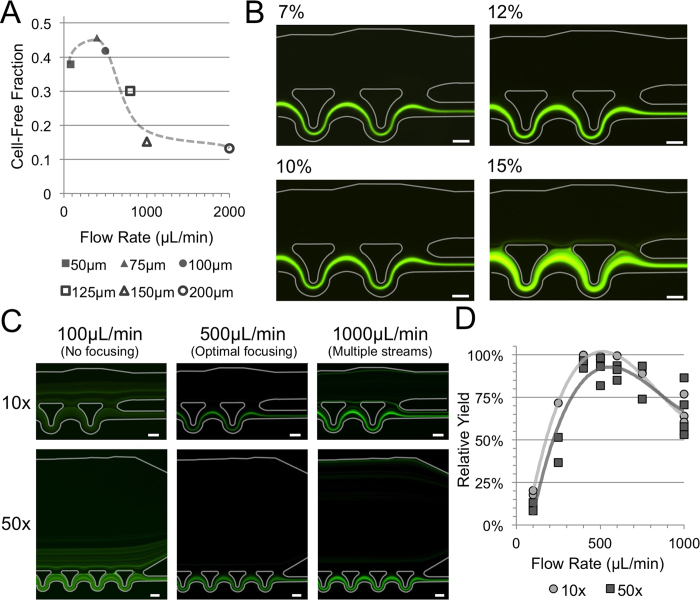
Relating Siphon Percentage and Flow Rate: (**A**) Plot of the maximum cell free fraction for different width devices plotted versus the flow rate at which the maximum cell free fraction is achieved. The thickness is given as a percentage of the channel width at a point downstream of the focusing units. (**B**) Fluorescent streak images of labeled buffy coat run through different 10x concentrator devices that were designed with increasing siphon percentages from top to bottom showing the loss of cells at the higher siphon percentages. (**C**) Fluorescent streak images of the 10x and 50x devices operating at different input flow rates increasing from left to right. The loss of cells at 100 μL/min is due to the lack of inertial focusing forces at the lower speeds. Optimal focusing performance is at the designed flow rate of 500 μL/min. The loss of cells at the higher flow rates can be seen at the top wall of the siphon channels where a well formed streak is located which exits the focusing channels at the first siphon. (**D**) The relative yield (# of cells in product / # of cells in product + waste) of the 10x and 50x devices with an input concentration of 1 M cells/mL at different flow rates. Scale bars are 50 μm.

Perhaps the most important design consideration is the control of the percentage of siphoned fluid relative to the dynamics of the cell free region formation. In inertial focusing systems the focusing behavior is a cumulative result of numerous parameters including the channel geometry as well as flow speed^22^,^29^. In previous comparisons of different geometries it has been concluded that curved structures are generally more efficient at achieving focusing over a given channel length while perhaps are more sensitive to changes in flow speed^30^. Using scaled versions of the asymmetrically curved structures previously detailed, we characterize the cell free region formation within a range of focusing channel widths from 50 μm to 200 μm over a large range of flow rates from 10 μL/min to 3000 μL/min depending on the channel width^17^. Each of these reference devices included a series of ten focusing units followed by an expansion into a 500 μm wide straight section. Using a solution of 9.9 μm fluorescent beads at a concentration of 1 × 10^6^ beads per mL buffer, the cell free region thickness was measured downstream of the focusing units after the channel had fully expanded using a 10% relative intensity threshold across the channel width^31^. The single sided cell free region thickness on the top (opposite the focusing channels) at the optimal flow rate for each channel width can be compared as shown in Fig. 2A. Images of these reference devices are available in the Supplementary Materials Figures 1–6. The narrower channels achieve significantly higher maximum cell free region thicknesses (50 μm wide - 38%, 75 μm - 46%, 100 μm - 42%, 125 μm - 30%, 150 μm - 15%, 200 μm - 13%) but as expected are more sensitive to flow rate as seen by the sharp peaks shown in Supplementary Materials Figure 7. The variation in cell free region thickness over a range +/− 50% of the optimal flow rate (flow rate which achieves the maximum cell free region thickness) was lower for the wider channels (50 μm wide - 12%, 75 μm - 23%, 100 μm - 16%, 125 μm - 15%, 150 μm - 4.6%, 200 μm - 5.5%).

Using the inertial focusing reference data we find that there is a nearly linear relationship between the optimal flow rate in μL/min, *Q*_*Optimal*_, (maximal cell free region formation) and the focusing unit width in μm, *w*_*focus*_ = 0.10911 * *Q*_*Optimal*_ + 44.789 plotted in Supplementary Figure 7. Using this relationship we can now create a device that maintains a high level of cell free region formation efficiency even as fluid is siphoned and the flow rate through the focusing channels decreases. In order to relate the cell free region formation to a maximum siphon percentage, a set of devices was designed using a range of siphon percentages (7%, 10%, 12% and 15%) at a fixed input flow rate of 500 μL/min, chosen to be well within the optimal flow rate range of the narrower more efficient focusing unit widths. A comparison of the focusing performance of these devices indicates that depending on the volume reduction factor desired and the lowest flow rate through the focusing channels near the outlet of a device, the siphon percentage must be below 10% for a 10x volume reduction and 7% for a 50x volume reduction device. A visual comparison of four different siphon percentages on a 10x device is given in Fig. 2B where the loss of cells into the top outlet in the 15% siphon percentage device is quite noticeable. The difference in optimal siphon percentage between the 10x and 50x devices is a consequence of an imposed minimum focusing channel width (50 μm focusing unit width - fabrication limitation). Any focusing units after this minimum width is achieved will no longer be creating an optimal cell free region. In the 50x device there are significantly more siphon units with this minimum width and therefore the lower siphon percentage mitigates the effects of less than optimal focusing.

From this point on, we acknowledge that the device performance is a complex variable mainly dependent on the complexities of the device geometry and therefore will select two specific designs for detailed characterization. The two selected designs that will be presented are a 10x concentrator (26 units, 10% siphon percentage) and a 50x concentrator (152 units, 7% siphon percentage) whose detailed design specifications are given in Supplementary Materials Figure 8.

### Flow Rate Dependence

Another significant factor in any inertial focusing system is the flow speed. While the devices shown here are specifically designed for an input flow rate of 500 μL/min the sensitivity to flow rate is important to investigate. Using isolated white blood cells (buffy coat), the relative yields of both the 10x and 50x devices were analyzed between 100 μL/min and 1000 μL/min. The device maintained high yield (>95%) between 400 and 600 μL/min but outside of this operational range the drop off in yield was significant as shown in Fig. 2C,D. Yield is calculated on a relative basis between the product and waste fractions or the total white blood cells in product divided by total white blood cells in the waste and product combined. In general, the system loss comparing the input concentration and volume processed to total cells coming out of the product and waste combined was typically low, less than 10%. Viability of the concentrated cells in the 50x device was measured using propidium iodide finding 94.2% (n = 3) of the cells to be viable after processing.

For the flow rates lower than 400 μL/min, the drop off in yield is consistent with an overall lack of focusing. In the case of negligible inertial effects, one would expect a yield equivalent to the flow split, 10% and 2% for the 10x and 50x devices respectively. The increase in yield by increasing the flow rate from 100 to 400 μL/min is indicative of the improvement of focusing with Reynolds number as inertial effects increase. The decrease in yield after 600 μL/min is a likely a consequence of PDMS deformation at the higher driving pressures leading to significantly different focusing patterns as shown in the streak images of Fig. 2C where there is a well focused stream of cells lost at the 1^st^ siphon position simply due to an equilibrium position forming at the wall closest to the siphons as the PDMS expands. The formation of this streak is consistent with the reference focusing device data at higher flow rates.

### Size Dependence

**Figure 3:**
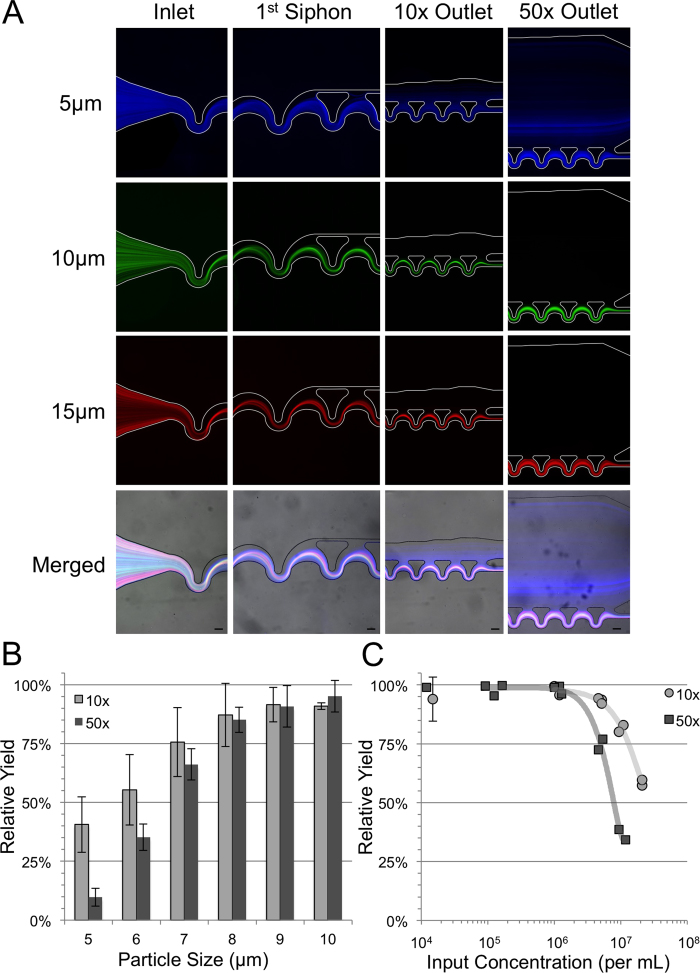
Particle Size and Concentration Dependence: (**A**) Fluorescent streak images showing the particle size sensitivity of the 10x (left) and 50x (right) devices. The particle diameter increases from the top row (4.4 μm blue) to the second row (9.9 μm green) to third row (15 μm red) and merged together with brightfield in the bottom row. Scale bars are 50 μm. (**B**) The size sensitivity of the relative yield through the 10x and 50x devices tested using discrete sizes of polystyrene particles. Error bars indicate plus/minus one standard deviation. (**C**) A plot of the relative white blood cell yield for the 10x and 50x devices as a function on input concentration showing the sharp drop off around 1 M cells/mL. All input concentrations below 20,000 cells per mL are grouped (N = 63 for 10x and N = 3 for 50x). Error bars indicate plus/minus one standard deviation.

Inertial forces are strongly dependent upon the size of the particles being focused and thus the performance of these devices must be evaluated to understand the sensitivity to particle size. Fluorescent images of three different particle sizes are shown in Fig. 3A for both the 10x and 50x devices. Both of the larger diameter particles, 15 μm (red) and 10 μm (green), show minimal loss over an extended exposure streak image (~1 s or 5*10^5^ events). The smallest 5 μm (blue) diameter particles, however, show significant loss to the top outlet. In order to get a better understanding of this particle size sensitivity, a variety of polystyrene particle sizes (4 μm–10 μm) at equal concentrations (100,000 beads/mL) were run simultaneously through the 10x and 50x devices in order to determine the size range that will be deflected in each device. As predicted, a general trend of smaller particle sizes having lower yields is apparent in the data shown in Fig. 3B. A cutoff size can be interpolated for both devices as the size at which the yield decreases below 90% which is found to be ~8.5 μm for the 10x device and 8 μm for the 50x. This slight difference is attributed to the significantly lower velocities at the end of the 50x concentrator where the focusing becomes more sensitive to particle size along the device after reaching the minimum focusing channel width at unit number 28 out of 153 as compared to 23 out of 26 for the 10x device. This size dependence can be beneficial for cleanup of biological samples as particles smaller than a cutoff size will be siphoned off improving the final sample purity or decreasing bacterial contamination. For instance, if bacteria follow the streamlines of the flow, 90% of the contaminating bacteria would be siphoned away using a 10x concentrator and 98% using a 50x concentrator. The same can be said for the use of this device for sample washing steps; as a sample is processed through the devices only a small fraction of the stain or original solution will still be in the produced concentrated sample.

### Volume Fraction Dependence

One of the more mysterious aspects of inertial focusing is the effect of inter-particle interactions on the focusing behavior. The majority of inertial focusing devices have the strict requirement of low input concentrations in order to achieve high quality focusing^32^. The theoretical limitation is given by the limit of a continuous line of adjacently touching particles at the equilibrium positions along the entire channel length, or in other words, a length fraction of 1^29^. We investigated the operational cutoff of the particle concentration for the 10x and 50x devices by varying the input concentration of white blood cells processed at 500 μL/min. The relative yield plotted in Fig. 3C at the different input concentrations indicates a sharp maximum input concentration limit at approximately 1 M cells per milliliter. The slight difference in this critical concentration between the 10x and 50x devices at which the yield drops off is indicative of the fact that a particle concentration threshold reached in the devices of approximately ~80 M cells per milliliter at which the particle interactions will start significantly affecting the performance of the device. This particle concentration is approximately a particle volume fraction of 4.18% or length fraction of ~4. This cutoff should ideally occur at an input concentration 5 times lower in the 50x device but is adjusted to a factor of approximately 2 considering the difference in siphon percentages between the designs along the devices.

At such high particle volume fractions, it is likely that the resistance calculations utilized in the design of the volume reduction devices are no longer accurate due to the increase in viscosity associated with an increase in particle volume fraction. High-speed videos showing the decreased relative velocity within the focusing channels are available in the Supplementary Materials (10x at 5 × 10^6^ per mL input concentration SI Figure 10 and 50x at 5 × 10^6^ per mL input concentration SI Figure 11). A design taking into account the increase in effective viscosity of the sample at higher particle volume fractions is also plausible but will be significantly dependent on the input sample concentration thus limiting its utility. One manner in which the increased viscosity could be taken into account is using a form Einstein’s effective viscosity relationship for hard spheres which posits that the effective kinematic viscosity increases with the volume fraction of hard spheres, η = η_0_(1 − 1.35φ)^−5/2^ 33.

### Achieving Greater Than 50x Volume Reduction

**Figure 4:**
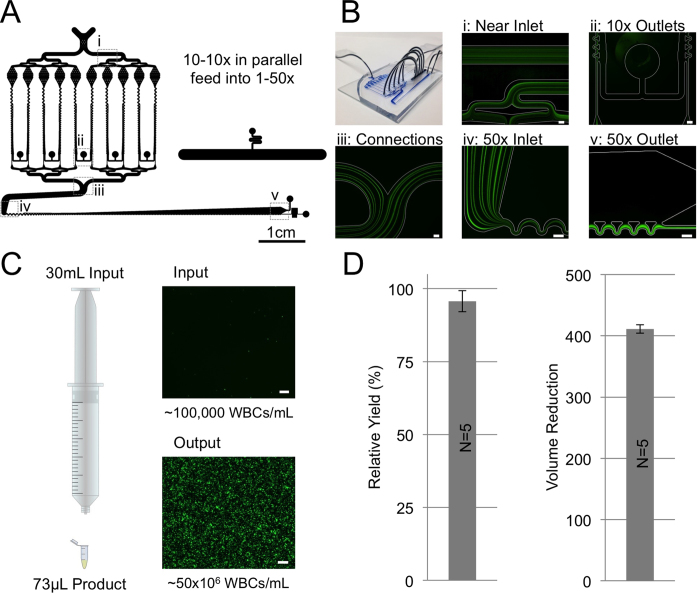
Serially Integrated 500x Device Design and Performance: (**A**) Schematic of the 500x device labeled with imaging positions. (**B**) Image of the device and fluorescent streak images of the device in operation showing (i) particles entering the device dispersed, (ii) a pair of 10x device outlets, (iii) ten joined streams of particles from the first stage of 10x devices, (iv) entrance of the 50x device where the ten stream become one and (v) the outlet of the 50x device. (**C**) Schematic representation of the typical input sample and output samples sizes with associated fluorescent images from one sample run. (**D**) The actual volume reduction factor measured as well as the relative yield over 5 different sample runs. Error bars indicate plus/minus one standard deviation. Scale bars are 100 μm unless otherwise noted.

An important consideration in the application of microfluidic volume reduction devices is the ability to surpass or standardize techniques used in everyday experiments. While a throughput and volume reduction factor of 500 μL/min and 50x respectively are already significant one could argue that a well-trained laboratory technician could achieve consistently greater results using standard centrifugation and aspiration. Therefore, in pursuit of higher throughput and volume reduction factors a serially integrated device was constructed using ten parallel 10x devices that feed into a single 50x device shown schematically in Fig. 4A as well as the operation of the device is shown using fluorescent streak images at several key positions along the device. Briefly, dilute particles enter the device (i), are focused in the separate 10x concentrators (ii) sending ten parallel focused streams through a series of converging channels (iii) which are then refocused as they enter the 50x device (iv) and finally all the particles exit through the bottom product outlet of the 50x device (v). Due to the pressure requirements and PDMS deformation, the devices were fabricated in rigid epoxy^31^,^34^. The yield of this integrated device was consistently above 95% for white blood cells (when below the input concentration limit of 100,000 per mL) and achieved a volume reduction factor of ~411. The discrepancy between the 411 factor and 500 designed factor is a difference of only a few microliters of product which was difficult to control as the input flow rate of 4 mL/min (pump driving force limitation) and the product flow rate of <10 μL/min. A fabrication imperfection in the rigid devices can alter this balance as well. Overall, as shown in Fig. 4C and D, a 30 mL input sample containing 100,000 white blood cells per mL will be reduced into 73 μL +/− 1.2 μL (n = 5) with greater than 95% of the original cells (95.7% +/− 3.6%, n = 5).

### Highlighted Applications

The utility of the presented inertial focusing siphon concentrators and the versatile nature of the design concept can be highlighted by three processes, summarized in Table 1, across which we now use the concentrator devices on a daily basis. The first application for the concentrator is for improving the blood processing method for leukocyte imaging^35^. In this process blood is drawn from a patient and white blood cells are isolated using a deterministic lateral displacement device. The WBCs are then concentrated to a smaller volume using a 10x concentrator and radiolabeled without the sterile breaks of typical centrifugation process and with improved RBC and platelet removal as compared to bulk techniques. This stained sample is then concentrated once more by 10x to remove any excess radioactive label and can then be directly reinjected into the patient to determine the extent and location of infections. The entire process takes less than 3 hours for processing 40 mL of whole blood while retaining ~80% of the leukocytes from the initial whole blood sample leading to a 1.3 times increase in imaging signal over conventional processing. The activation of leukocytes was not significantly different between using the microfluidic concentrator as compared to a low speed centrifugation, shown in Supplementary Figure 12.

The second application incorporates three separate concentrating devices for expanding the utility of a circulating tumor cell detection and discovery assay. In this procedure, CTCs are isolated using our iChip technology^9^,^36^ and then concentrated to a small volume (~220 μL) which enables running the entire sample through an imaging flow cytometer (Amnis, Seattle Washington) gathering size, surface marker and nuclear morphology information. While this is typically an endpoint for experiments due to the high dilution factor of the sheath flows within the cytometer (~400 times or an 88 mL final volume) the output can instead be reconcentrated (10x and 50x) to a volume smaller than the original sample (~176 μL) and reused for molecular assays such as Sanger sequencing for detecting specific genetic mutations.

The final application exemplifies the most versatile aspect of the concentrator devices: the ability to integrate large samples with low flow rate microfluidic technologies. In this example, a 50x concentrator with an output of ~10 μL/min directly feeds into a non-centrifugal plating chamber coated with poly-d-lysine, used for enhancing cellular adhesion. Using secondary inlet ports, automated staining of the cells can be accomplished within the chamber after concentrating with minimal cell loss, ~1%. The output flow rate of the concentrated product is in the operational range of many other types of microfluidic technologies that could benefit from having an upstream concentration step such as automated cell staining^37^, cell trapping^38^,^39^, cell encapsulation^40^, buffer exchange^10^ and sorting technologies^12^. These possibilities illustrate the main advantage of these devices to provide a tailored macro-sample to microfluidic interface enhancing the utility of microfluidic devices.

## Discussion

Most laboratory procedures involve multiple steps of volume reduction or centrifugation, which are easily replaced with the microfluidic inertial focusing siphoning devices presented here. We show that the microfluidic method can concentrate bioparticles in a continuous flow manner at a throughput of up to 4 mL/min (240 mL/hour) and achieving a volume reduction factor of greater than 400. The conceptual design takes advantage of fast-acting inertial forces, which generate a cell free region near the walls of the channel. This cell free fluid region is then controllably siphoned off leaving the cells once again closer to the walls where the forces are strongest. The process of focusing and siphoning is repeated until a desired volume reduction is achieved. The operation of a set of the volume reduction devices is validated using buffy coat samples illustrating the high yield, >95%, and reproducibility of the device operation. The inertial focusing based microfluidic process presented exposes each cell passing through the device to equivalent conditions and only for ~100 ms. The device operation is dependent upon particle size rather than particle density thus making it unique compared to size and density based centrifugation. The microfluidic concentrator overcomes the undesirable features of centrifugation, which involve significantly longer exposure of cells to highly non-uniform stresses, lossy processing, multiple samples for large volumes, and packing of the cells into a pellet.

Inertial focusing has been mentioned as a means of enriching bioparticles based upon size^25^, shape^19^,^20^ and deformability^26^,^27^. However these studies and devices were tailored for separating particles rather than concentrating them. The few specifically presented concentrator devices either utilized a single flow split at the outlet of the device^41^ or intermittent removal of the cell free fluid a few times along the outer turns of a spiral device^42^. The highest achieved volume reduction factor has been only ~15 in such devices, well below what is achieved in a typical centrifugation process^28^,^42^. Other deterministic lateral displacement devices have been shown to achieve up to 40x concentration factors using larger cancer cell lines at high throughput up to 10 mL/min, however, this throughput would likely be decreased when the dimensions are tailored for use with smaller white blood cells^43^,^44^,^45^. Compared to these other microfluidic devices, our inertial focusing siphon devices achieve a higher per channel concentration factor at similar throughput. We also show an increased particle concentration cutoff of 80 M cells/mL that is significantly higher than the critical concentration given by Di Carlo for inertial focusing^29^. We attribute the high capacity of microfluidic inertial flow siphoning device to concentrate to the fact that the operational success or yield of the siphon devices no longer requires that all of the particles fall on a single streamline as they are concentrated given that we are taking advantage of the cell free region formation near the walls to siphon while the cells are being concentrated^29^. Despite this increased cutoff the device is more suitable for concentrating initially dilute samples such as rare cell populations. Possible design improvements for extending this utility could involve another siphon channel which would be designed contain the higher volume fraction material which the focusing units would work at the more interface between the cell rich and cell free fractions as well as the aforementioned idea of correcting the design program for the dependence of viscosity on volume fraction.

The applicability of the inertial focusing siphoning concept can also be expanded beyond the presented samples to smaller particles such as bacteria and fungi. In fact there is evidence in the literature for the applicability of inertial focusing for particles as small as 2 μm^30^. Essentially, the same design process developed and presented here can be applied to other particles and even device geometries by first obtaining reference focusing data and then applying the same siphoning design program. The asymmetrically curved structures here are given as a good example of one type of geometry that can be used in this manner. For smaller target bioparticles, the channel dimensions must be smaller in order to focus the smaller particles thus decreasing the single channel throughput but as long as the bioparticles focus the siphon design scheme presented here can be applied. The shape of these smaller bioparticles may have an effect on the performance of a given concentrator design but according to previous results^46^, the focusing behavior depends on the largest diameter of the particles. This potentially expands the clinical opportunities of the device to fungi and bacteria with a broad range of potential applications in infectious disease diagnosis at the point of care or resource limited regions.

There are an even greater number of possibilities for the technology when considering that while we have presented a single device which achieves a throughput of 500 μL/min at a volume reduction factor of 50x, there are simple ways of parallelizing these channels into a set of greater than 40 (20 mL/min or 1200 mL/hr), diminishing the run time for larger clinical samples and increasing the concentration factor to 2500^47^. This enables a new realm of possible applications as there are numerous examples of large volume bodily fluids and important clinical samples which cannot be efficiently processed in standard centrifuges. These include peritoneal washings^48^ and ascities^49^ where the sample volumes are of the order of 100 mL to 1 L and extremely dilute cells are of high clinical significance for several types of cancer and infections. These figures for improved throughput and performance do not include the advantages associated with the transition from PDMS to rigid materials (smaller footprint required and higher throughput per channel).

The ultimate utility of the microfluidic volume reduction devices is dependent upon its versatility and ease of integration with typical laboratory processes. The three applications highlighted here encompass the several key advantages of the technology and show its utility in the manipulation complex bodily fluids as well as large volumes of fluids. By removing sterile breaks associated with centrifugation from the process for isolation and radiolabelling of leukocytes, a fully automated and sterile process can be developed using the microfluidic inertial focusing siphon concentrator. The devices also can easily be made part of laboratory processes as shown with the multiple devices integrated into a CTC isolation process allowing for more information to be collected from the same rare cells. Finally and perhaps most importantly, the ability to use the inertial focusing siphon concentrator device to directly couple large volume biological samples with the continuously increasing number of microfluidic technologies.

In conclusion, we have developed a high-throughput microfluidic analog to macroscopic or bulk centrifugation commonly used for reducing the volume of dilute bioparticle suspensions. The microfluidic method is capable of reducing the volume of a dilute sample in a controlled manner up to 400 times in a continuous flow manner. We believe that the adaptability and ease of operation of these systems make it feasible to integrate into point-of-care devices with broad range of applications in diagnostics and global health.

## Methods

### Device Design and Optimization

The design procedure for the presented devices involves two major steps. First, reference devices with asymmetrically curved microchannels of different widths are tested over a wide range of flow conditions using exemplar particles, in this case 10 μm fluorescent polystyrene beads and white blood cells as shown in Supplementary Materials Figures 1–6. The cell free region is analyzed across these conditions using the fluorescent intensity across the channel cross section at the same position in each set of reference devices. The cell free region is then plotted versus flow rate and an optimal flow rate is chosen for each width device (Supplementary Materials Figure 7—fluorescently labeled buffy coat samples diluted to 1 × 10^6^ cells/mL). The optimal flow rate is chosen as the midpoint between the two flow rates that achieve 90% of the maximum cell free region thickness.

This leads to the second step of the process which is the hydraulic resistance balancing which is at the heart of controlling the amount of fluid siphoned in each unit along the channels. A set inlet flow rate of 500 μL/min was chosen as for higher flow rates and wider channels the cell free fraction decreases. After 5 units at the optimal size for this flow rate (99 μm wide) the first siphon unit is designed to remove an amount of fluid based upon a percentage of the flow through the focusing units. This siphon percentage, or percent of the volumetric flow rate through the focusing channels that is siphoned in each unit, is kept uniform (exponentially decreasing flow rate through each consecutive siphon gap). The width of the side channel is controlled to be a certain hydraulic resistance relative to the focusing channel thereby controlling this balance. A minimum width of 50 μm for the focusing channel was required as the focusing behavior within such channels is strongly dependent of aspect ratio of the channel^50^. Once this minimum size is reached (focusing channel flow rate of 47.76 μL/min) the siphon percentage is controlled solely by the side channel expansion, rather than by the combined effect of changing focusing channel size and side channel width. A factor of safety is implemented such that a device designed to achieve 10x concentration is extended until a theoretical value of 15x is reached and then the flow split is controlled using hydraulic resistors on the outlet product and waste channels. As previously stated, with greater understanding of the designs it is possible to remove this factor of safety in order to reduce the footprint of the device.

Another important consideration is that since a smallest width is imposed the focusing efficiency will diminish as the flow rate in the focusing channel decreases in the later units in the device. This is counteracted by a linear decrease in the siphon percentage relative to the decrease in the average velocity of the flow in the focusing channel after the minimum width is achieved.

### Hydraulic Resistance Calculations

The resistance is calculated using a classical definition of the Hagen-Poiseuille flow resistance in a rectangular cross section channel^51^. A 20-term sum of the Fourier series is completed using a custom MATLAB code to get the resistance for any straight section of channel with a length of l, height of h and width of w using a dynamic viscosity that of water or 0.001003 Pas. Non-uniform width sections of the channel are calculated as 1000 distinct equal length sections of channel of linearly increasing width between the initial and final widths of that section. The resistance of the focusing unit is always assumed to be equal to that of the closed (reference units) or non-siphoning unit, in other words, ignoring the opening between focusing and siphoning units.

### Fabrication

Standard SU8 photolithography and soft lithography were used to fabricate the master mold and the polydimethylsiloxane (PDMS) microchannels respectively. Briefly, negative photoresist SU8-50 (Microchem Corp, Massachusetts) was spun at 2850 RPM to a thickness of approximately 52 μm (channel depth kept constant between all devices +/− 2 μm), exposed to UV light through a mylar emulsion printed photomask (Fineline Imaging, Colorado) and developed in BTS-220 SU8-Developer (J.T. Baker, New Jersey). A 10:1 ratio mixture of Sylgard 184 Elastomer base and curing agent (Dow Corning, Michigan) was then poured over the raised mold, allowed to cure in an oven at 65 °C for 8 hours and then removed from the silicon/SU8 master. Inlet and outlet holes were punched using custom sharpened needle tips. The devices were then cleaned of particulate using low-residue tape and oxygen plasma bonded to pre-cleaned 1 mm thick glass microscope slides.

Epoxy devices were constructed using PDMS molds created by treating PDMS channels with tridecafluoro-1,1-2,2-tetrahydrooctyl-trichlorosilane (Gelest) and then pouring PDMS over the silanized channels. After 24 hours of curing at 65C the molds are carefully separated from the silanized channels. Holes were punched into PDMS molds at the inlets and outlets using a 0.75 mm diameter Harris Uni-Core biopsy punch. Teflon coated wire (0.028 inch diameter, McMaster-Carr) was inserted gently into these holes as to not deform the surface of the PDMS mold. Tygon tubing (.02” I.D., .06” O.D.) was then guided onto teflon coated wire and suspended ~1 mm from mold surface. Epoxacast 690 (Smooth-On) was mixed and degassed for 30 minutes prior to pouring into the mold. At the same time as molds were filled, slides were coated with epoxy by laying a glass slide on a drop of epoxy atop a flat PDMS surface. After ~28 hours devices were cooled temporarily to −22 C to prevent deformation, the Teflon wire was removed and devices removed from the molds. Then the glass slides were removed from the PDMS slabs and heated to 55 C and devices were pressed against slides ensuring bonding.

### Particle and Cell Suspensions

Polystyrene particle suspensions were created using 4.4 μm blue-fluorescent beads (Polysciences), 9.9 μm green-fluorescent beads (ThermoFisher Scientific) and 15 μm red-fluorescent beads (Invitrogen). Each was suspended to a final length fraction of 0.1 in an equivalent density solution (1.05 g/mL) of 1x PBS, 0.1% Tween20, and iodixanol. White blood cells (buffy coat) were isolated using deterministic lateral displacement with a co-flow of buffer which has been previously published^9^.

### Cell Counting

Hemocytometers and Nageotte chambers were utilized for measuring particle concentrations in white blood cell yield experiments at dilutions dependent upon the output cell concentrations.

### Fluorescent Imaging

Fluorescent and high imaging was accomplished using an automated Nikon TiE inverted microscope with a Retiga 2000R monochromatic camera as well as a Vision Research Phantom v4.2 high speed monochromatic camera.

## Additional Information

**How to cite this article**: Martel, J. M. *et al.* Continuous Flow Microfluidic Bioparticle Concentrator. *Sci. Rep.* **5**, 11300; doi: 10.1038/srep11300 (2015).

## Supplementary Material

Supplementary Information

Supplementary Figure 11

Supplementary Figure 10

## Figures and Tables

**Table 1:** 

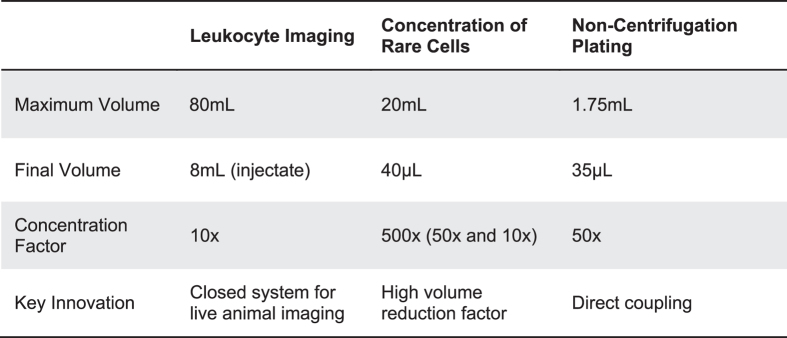

Summary of each of three highlighted applications of the microfluidic concentrator illustrating the wide utility.
