# Supplementary material for: Continuous Flow Microfluidic Bioparticle Concentrator
Source: Sci Rep. 2015 Jun 10;5:11300. doi: 10.1038/srep11300 (PMC4462155; doi:10.1038/srep11300)
Supplement: Supplementary Information [file srep11300-s1.pdf]

Continuous Flow Microfluidic Bioparticle Concentrator

Joseph M. Martel[1]*, Kyle C. Smith[1]*, Mcolisi Dlamini[1], Kendall Pletcher[1], Jennifer Yang[1], Murat Karabacak[1], Daniel A. Haber[2], Ravi Kapur[1] and Mehmet Toner[1,3]†

SUPPLEMENTARY MATERIALS

The following 6 figures are from the reference devices of 5 units of different focusing channel widths. Each is run at several flow rates, Q_{in}, to achieve equivalent channel Reynolds numbers, Re_C, in the different width channels. Re_C is defined here as $Re_C=3\rho U_{Avg}D_h/2\mu$, where ρ is the fluid density, U_{Avg} is the average velocity of the fluid, D_h is the hydraulic diameter of the focusing channel (D_h=2*height*width/(height+width)) and μ is the dynamic viscosity of the fluid.

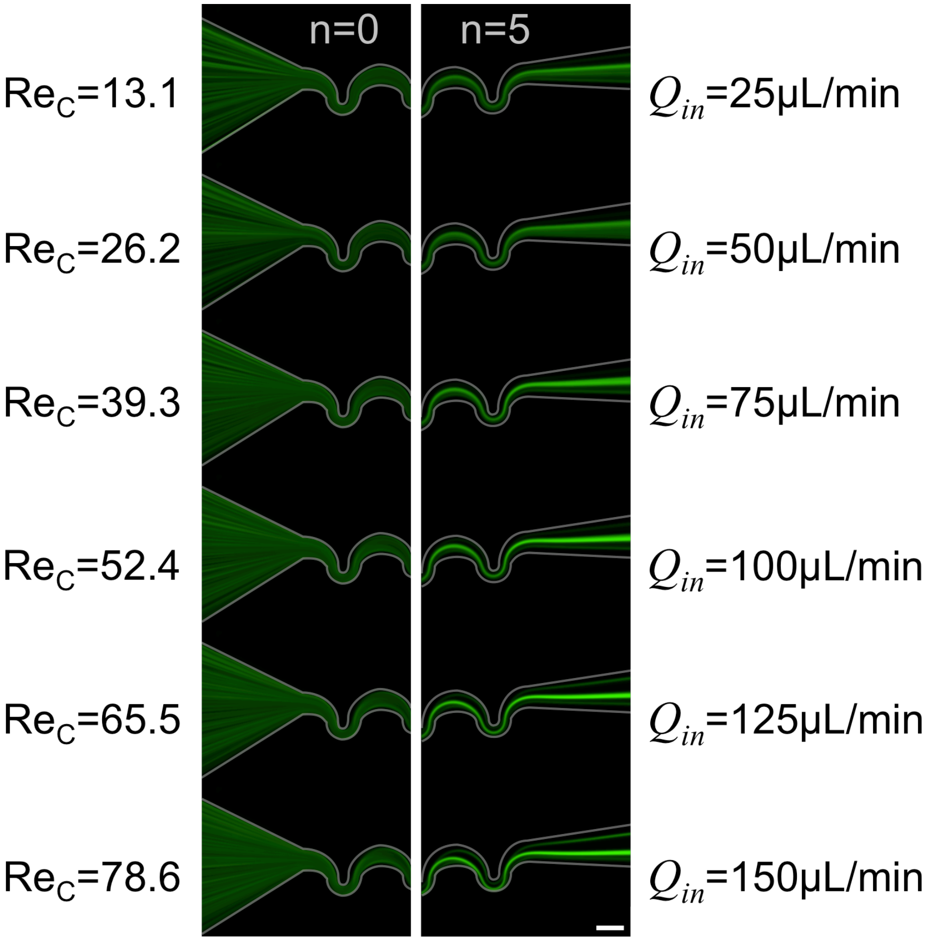

Supplementary Figure 1: Reference images using 9.9µm fluorescent beads for 50µm wide asymmetrically curved focusing channels. Images are taken at the beginning (left) and end (right) of the 5 focusing units at increasing flow rates from the top to bottom row. Scale bar is 100µm.

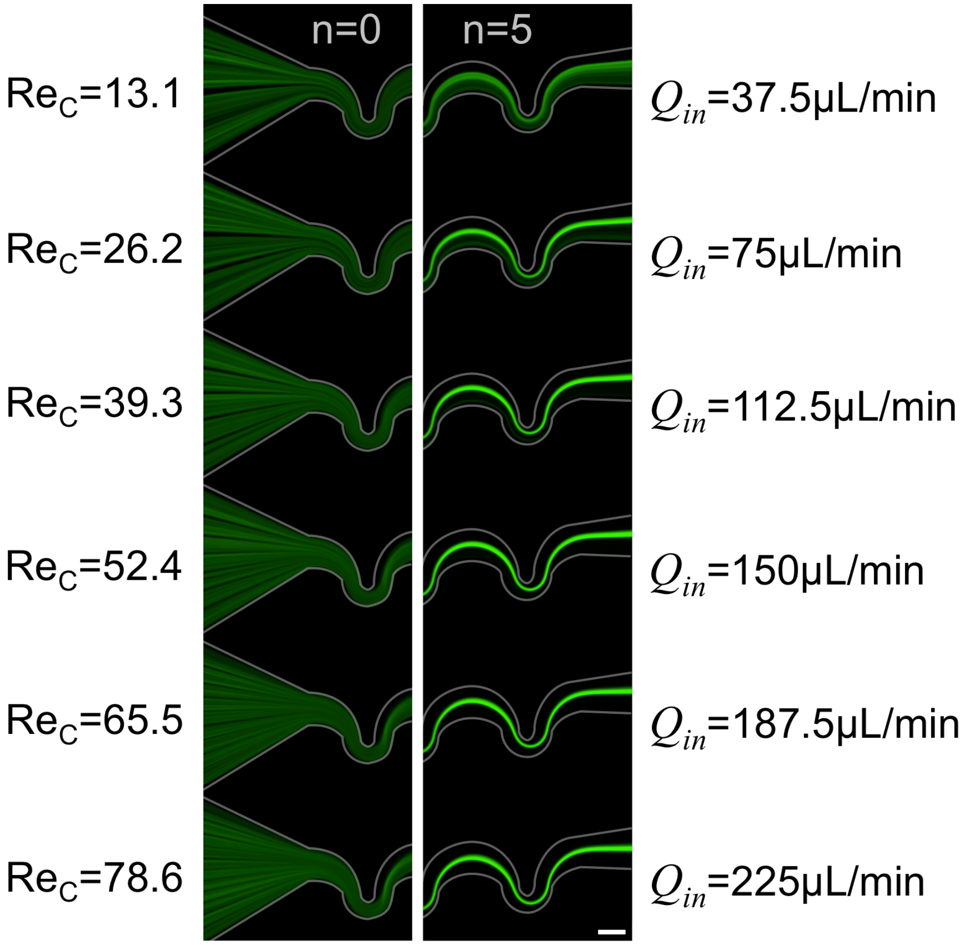

Supplementary Figure 2: Reference images using 9.9μm fluorescent beads for 75μm wide asymmetrically curved focusing channels. Images are taken at the beginning (left) and end (right) of the 5 focusing units at increasing flow rates from the top to bottom row. Scale bar is 100μm.

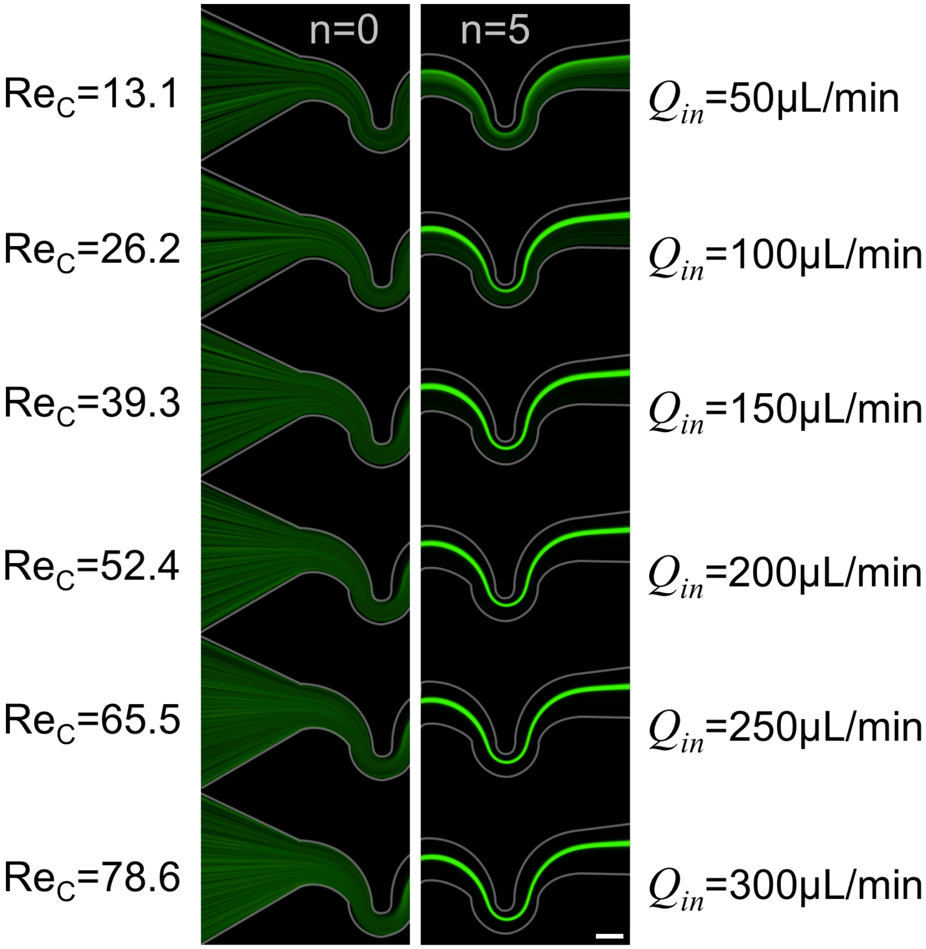

Supplementary Figure 3: Reference images using 9.9μm fluorescent beads for 100μm wide asymmetrically curved focusing channels. Images are taken at the beginning (left) and end (right) of the 5 focusing units at increasing flow rates from the top to bottom row. Scale bar is 100μm.

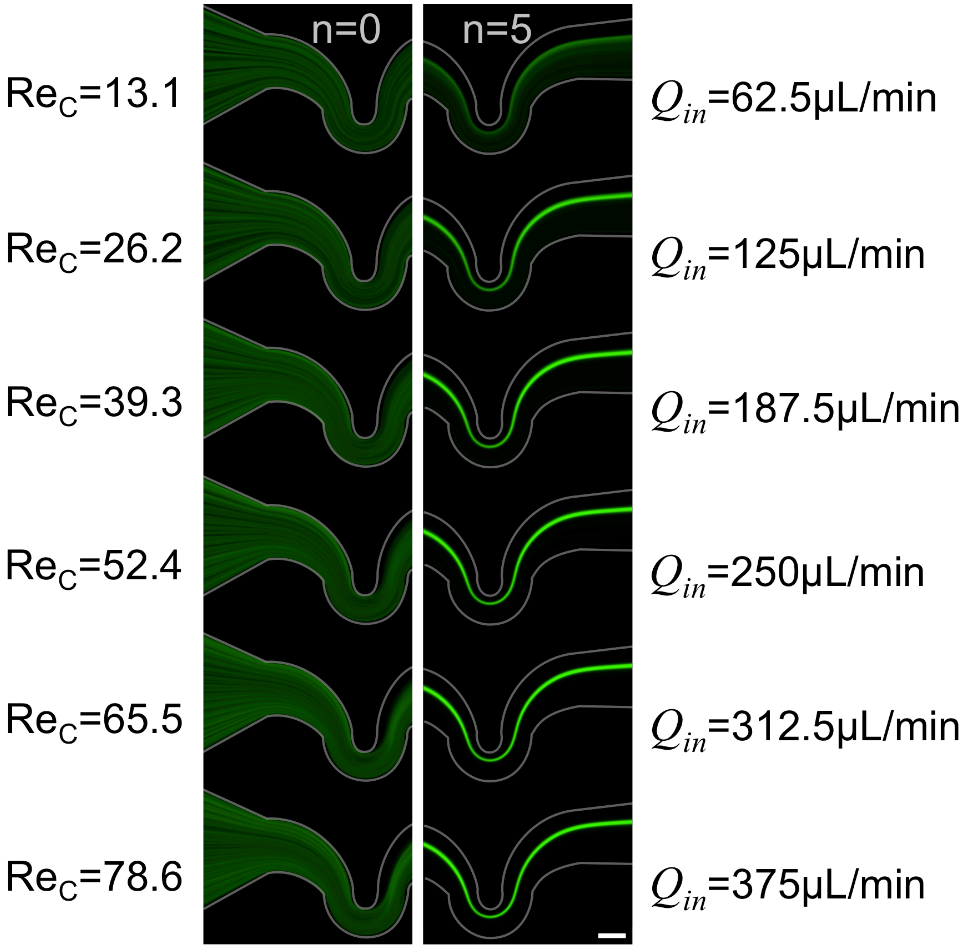

Supplementary Figure 4: Reference images using 9.9μm fluorescent beads for 125μm wide asymmetrically curved focusing channels. Images are taken at the beginning (left) and end (right) of the 5 focusing units at increasing flow rates from the top to bottom row. Scale bar is 100μm.

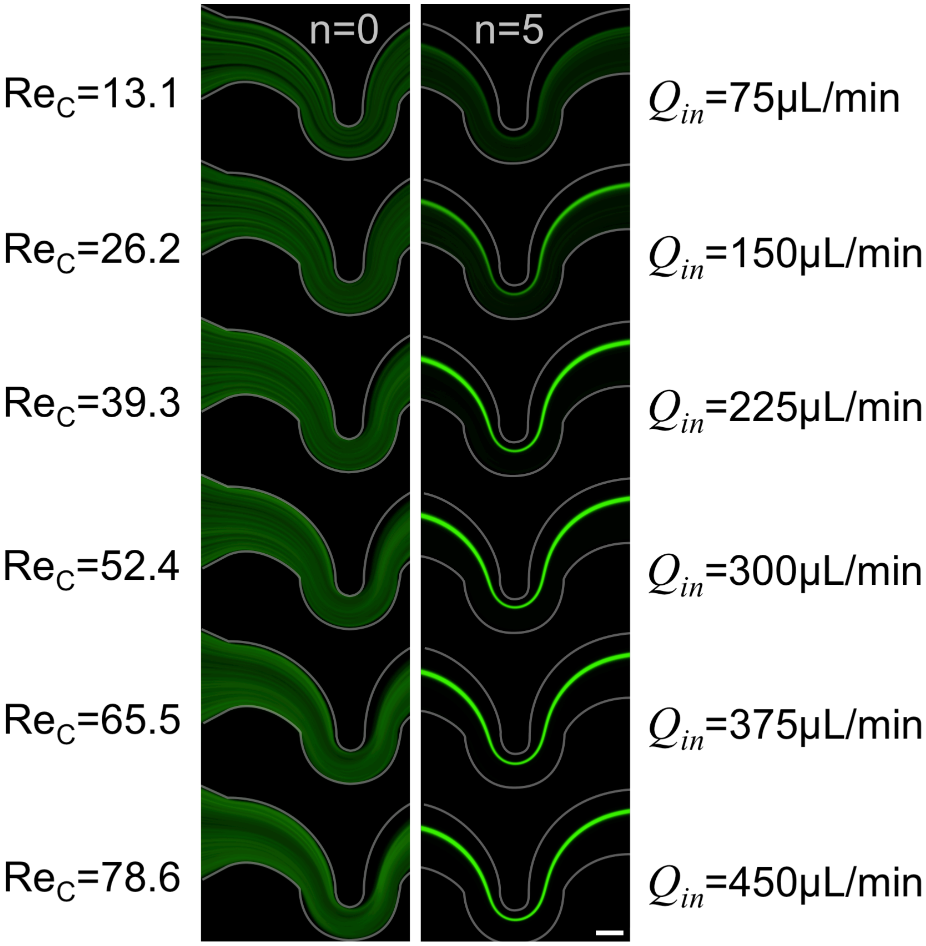

Supplementary Figure 5: Reference images using 9.9μm fluorescent beads for 150μm wide asymmetrically curved focusing channels. Images are taken at the beginning (left) and end (right) of the 5 focusing units at increasing flow rates from the top to bottom row. Scale bar is 100μm.

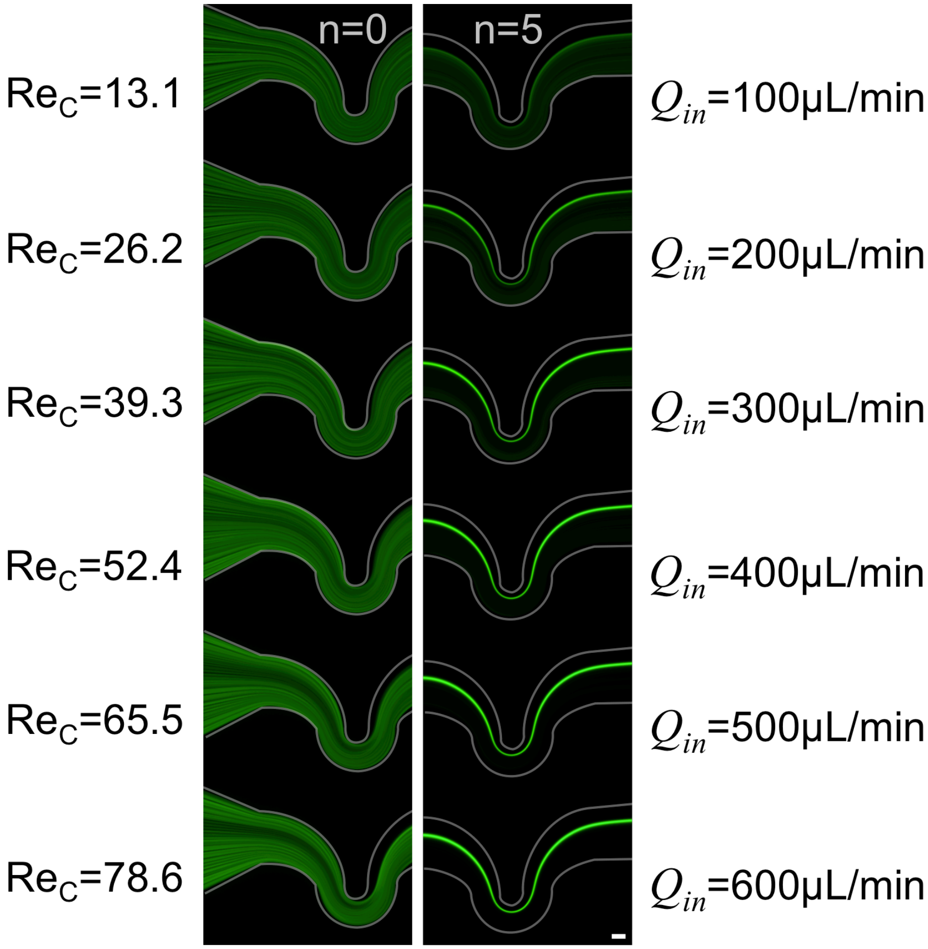

Supplementary Figure 6: Reference images using 9.9μm fluorescent beads for 200μm wide asymmetrically curved focusing channels. Images are taken at the beginning (left) and end (right) of the 5 focusing units at increasing flow rates from the top to bottom row. Scale bar is 100μm.

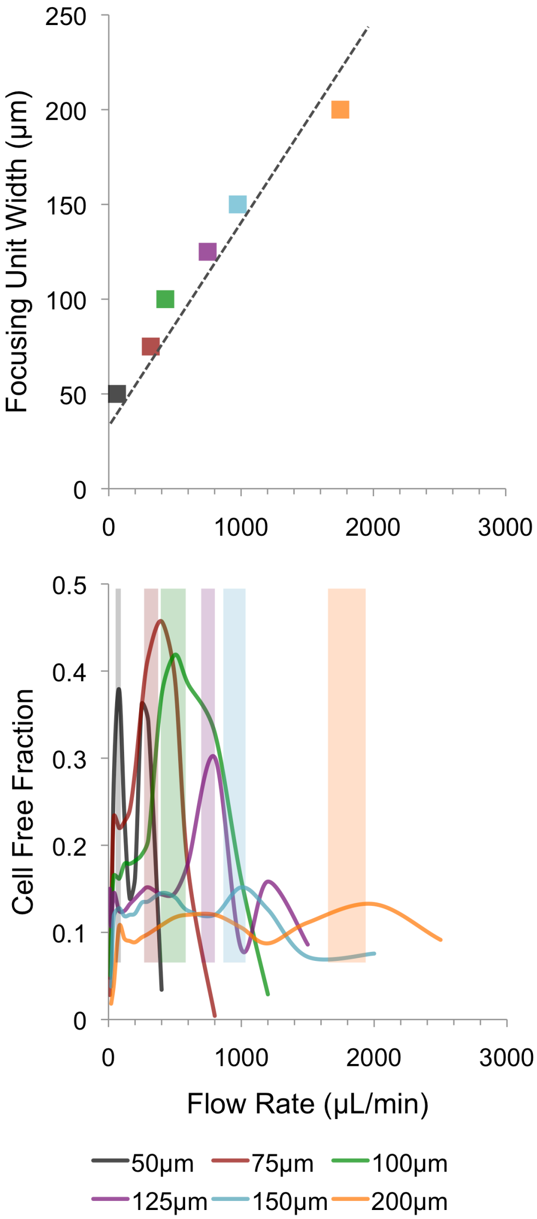

Supplementary Figure 7: (Top) Plot of optimal flow rate versus focusing channel width, defined as the flow rate at which the cell free layer is maximized in the reference devices. Data is collected using fluorescently labeled buffy coat diluted to 1M cells/mL of 1% F68 in 1X PBS buffer. (Bottom) Related reference data showing the cell free fraction relationship with the focusing channel width across different ranges of flow rates.

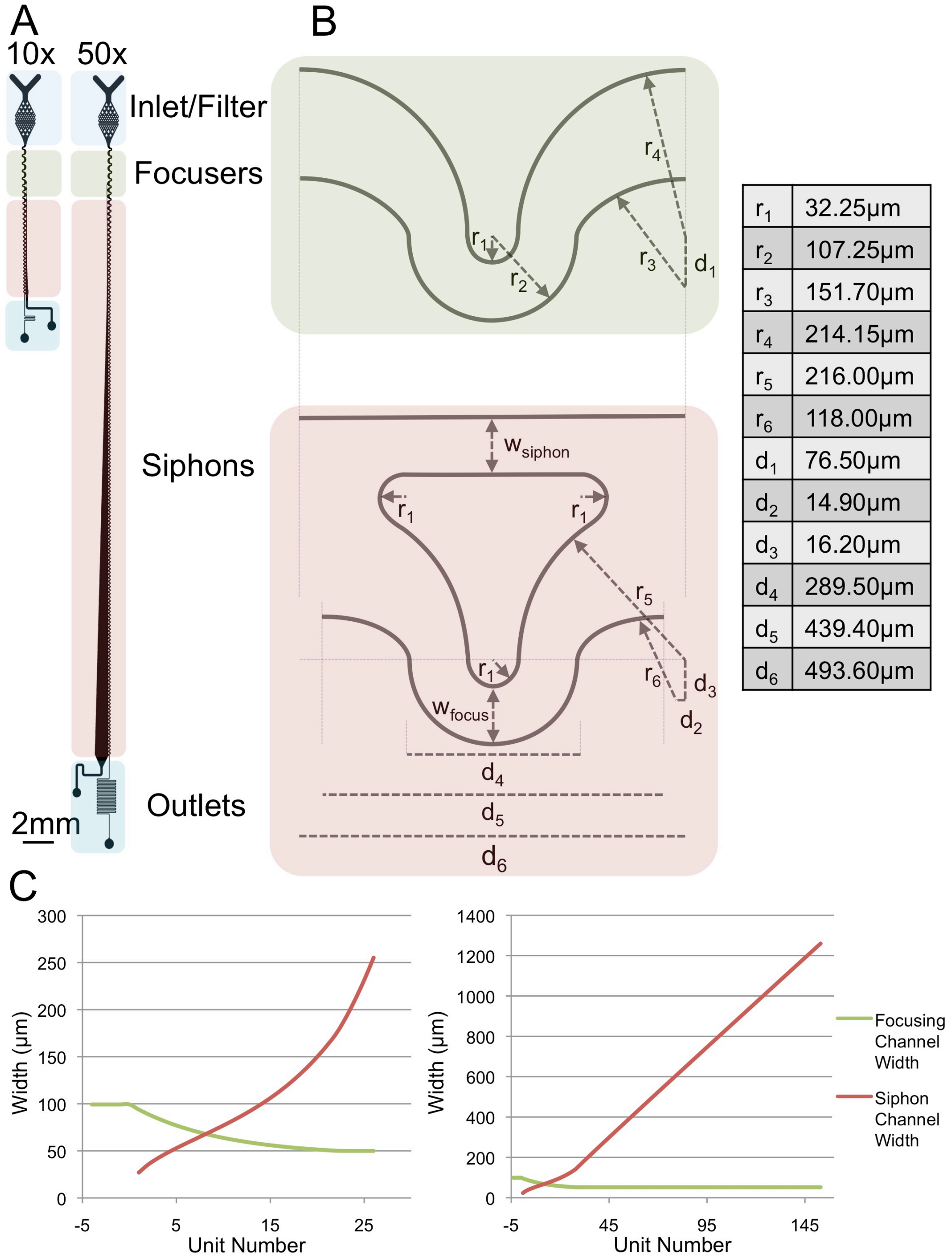

r_1	32.25µm
r_2	107.25µm
r_3	151.70µm
r_4	214.15µm
r_5	216.00µm
r_6	118.00µm
d_1	76.50µm
d_2	14.90µm
d_3	16.20µm
d_4	289.50µm
d_5	439.40µm
d_6	493.60µm

Supplementary Figure 8: (A) Schematics of the 10x and 50x volume reduction devices. (B) Dimensions of the two main units that are then scaled by the focusing channel width, w_{focus}: focusing channel without a siphon channel (top) and with a siphon channel bottom as well as the key dimensions. (C) The width of the focusing channel and side channel along each device given by unit for the 10x device (left) and 50x device (right). The channel depth is 52+/-2µm.

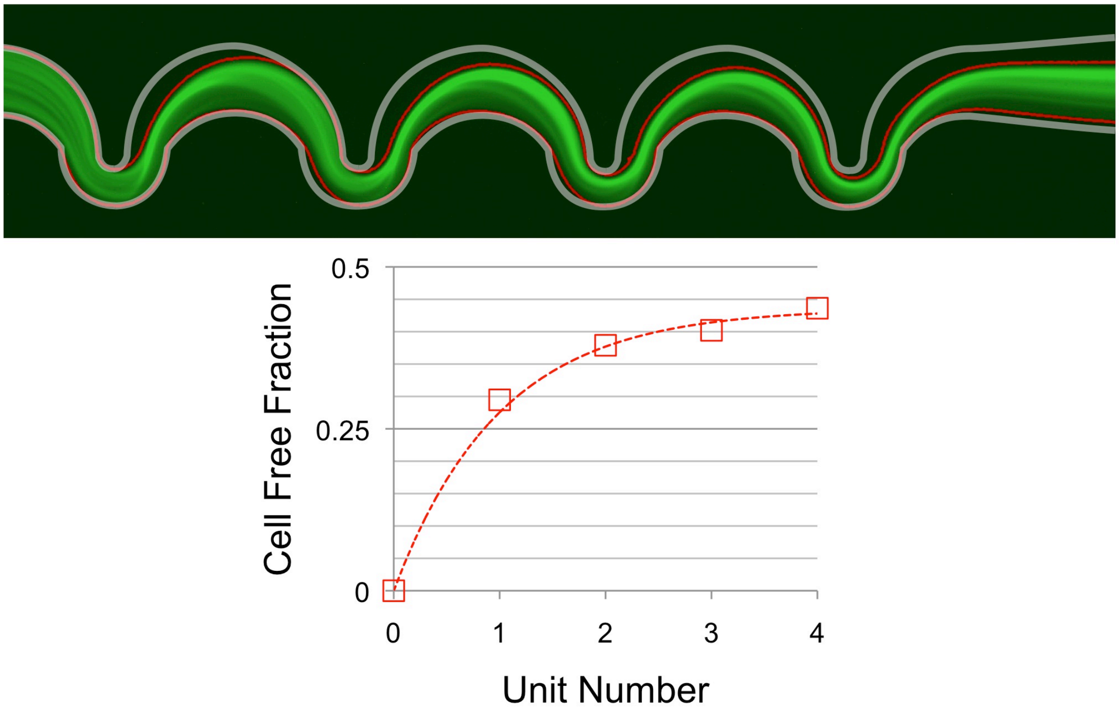

Supplementary Figure 9: Image of the cell free layer formation in a 100μm wide focusing channel across four focusing units. The red outline shows the 10% intensity threshold cutoff for determining the width of the particle distribution.

Supplementary Figure 10: (Movie) High-speed imaging results of the operation of the 10x volume reduction device at an input concentration of 5M white blood cells per mL at a flow rate of 500μL/min. Notice that near the outlet cells begin to be lost as the particles become too crowded within the focusing channels.

Supplementary Figure 11: (Movie) High-speed imaging results of the operation of the 50x volume reduction device at an input concentration of 5M white blood cells per mL at a flow rate of 500μL/min. Notice that near the outlet cells begin to be lost as the particles become too crowded within the focusing channels and the relative velocity of the cells within the focusing channel is noticeably different from those which are within the siphon channel likely indicating a different in effective viscosity due to particle volume fraction.

Leukocyte Activation Measurement: Relative activation of leukocytes was measured using imaging flow cytometry (Amnis Image Stream X) quantifying the CD11b expression of white blood cells exposed to different processing conditions. After concentration/exposure each sample was stained for 30 minutes with CD11b (AF388 Stemcell Technologies 60040AD – 2μL added to 400μL sample) and CD45 (PE Miltenyi Biotech 103-098-141 – 0.3μL added to 400μL sample) and 15 minutes with 1.25μM DRAQ5 (Life Technologies). A positive control was accomplished by exposing DLD isolated white blood cells to 0.2μM PMA (Sigma-Aldrich P1585 – phorbol 12-myristate 13-acetate) also known as TPA (12-O-Tetradecanoylphorbol-13-acetate), a potent agonist, for 30 minutes in an incubator and then stained. Isolated white blood cells were also diluted to 100,000 WBCs per mL and then concentrated using either a 50x inertial focusing siphon concentrator device or centrifugation in a 15mL conical at 125 r.c.f. for 5 minutes. Supplementary Figure 12 presents the relative average CD11b expression as compared to the average expression for the input WBCs isolated using DLD (only CD11b positive events are counted in the statistics). The only non-significant difference between the different test conditions was between the IFC and centrifugation conditions that were not significantly different (*p=0.0943).

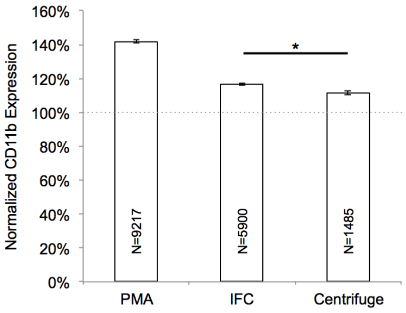

Supplementary Figure 12: Relative CD11b expression of CD11b positive events between different test cases. White blood cells isolated using deterministic lateral displacement (DLD WBCs), DLD WBCs treated with 0.2μM PMA for 30 minutes at 37C (PMA - positive control), cells concentrated using the microfluidic inertial focusing concentrator (IFC) and cells concentrated using a 5 minute centrifugation at 125 r.c.f. Population means are plotted with standard error

shown with error bars. All comparisons were significant ($p<0.0001$) except for the IFC and centrifugation conditions that were not significantly different (*$p=0.0943$).
